# Which anatomic structures are responsible for the reduction loss after hybrid stabilization of osteoporotic fractures of the thoracolumbar spine?

**DOI:** 10.1186/s12891-020-3065-3

**Published:** 2020-01-29

**Authors:** Ulrich J. Spiegl, Annette B. Ahrberg, Christine Anemüller, Jan-Sven Jarvers, Stefan Glasmacher, Nicolaus von der Höh, Christoph Josten, Christoph-Eckhard Heyde

**Affiliations:** Department of Orthopaedics, Trauma Surgery and Plastic Surgery, University of Leipzig, Liebigstr. 20, 20 04103 Leipzig, Germany

**Keywords:** Hybrid stabilization, Osteoporotic vertebral fracture, Reduction loss, Intervertebral disc, Thoracolumbar spine

## Abstract

**Introduction:**

Hybrid stabilization is an accepted therapy strategy for unstable osteoporotic thoracolumbar fractures. However**,** a moderate reduction loss has been reported and it remains unclear which anatomic structure is responsible for the reduction loss.

**Methods:**

This retrospective study was performed at a level I trauma center. Patients aged 61 and older were stabilized using hybrid stabilization after suffering acute and unstable osteoporotic vertebral body fractures at the thoracolumbar spine. Posterior stabilization was done short-segmental and minimal invasive with cement-augmentation of all pedicle screws. The minimum follow-up has been 2 years. The outcome parameters were the reduction loss and the relative loss of height of both intervertebral discs adjacent to the fractured vertebral body, the fractured vertebral body and a reference disc (intervertebral disc superior of the stabilization) between the postoperative and latest lateral radiographs. Additionally, implant positioning and loosening was analyzed.

**Results:**

29 mainly female (72%) patients (73.3 ± 6.0 years) were included. Fractures consisted of 26 incomplete burst fractures and 3 complete burst fractures of the thoracolumbar junction (Th11 – L2: 86%) and the midlumbar spine. The mean follow-up time was 36 months (range: 24–58 months). The mean reduction loss was 7.7° (range: 1–25). The relative loss of heights of both intervertebral discs adjacent to the fractured vertebral body, the reference disc, and the central vertebral body were significant. Thereby, the relative loss of the superior disc height was significant higher compared to the reference disc. Additionally, only the relative loss of central vertebral body height and reduction loss correlated significantly. There were no signs of implant loosening in any patient.

**Conclusions:**

The mean reduction loss was moderate 3 years after hybrid stabilization of unstable osteoporotic vertebral fractures of the thoracolumbar spine. A significant loss of both adjacent disc heights and the central vertebral body was seen, with the highest loss in the superior adjacent disc significantly outranging the reference disc. The superior adjacent intervertebral disc and the central part of the fractured vertebral body seem to be responsible for the majority of reduction loss.

## Introduction

Hybrid stabilization is an accepted therapy strategy for unstable osteoporotic thoracolumbar vertebral fractures leading to good clinical outcomes in the majority of the patients [1, 2]. The combination of cement augmentation of the fractured vertebral body and additional posterior stabilization helps to stabilize the fracture preventing severe malalignment. Trauma associated intervertebral disc lesions following osteoporotic vertebral fractures, mainly due to low-energy trauma, seem to be rather unlikely. Thus, relevant loss of intact intervertebral disc heights despite age-related sclerotic changes and additional posterior stabilization cannot be expected [1]. However, Spiegl et al. [2] reported a mean reduction loss of more than 7° after hybrid stabilization of osteoporotic vertebral fractures. It remains unclear which anatomic structures are responsible for the reduction loss. Is it caused by a reduction loss of the fractured vertebral body or is it localized at the adjacent intervertebral discs?

The aim of this study was to analyze the location of the reduction loss after hybrid stabilization of unstable burst fractures of the thoracolumbar spine in patients aged 60 years or higher. Our hypothesis was that the majority of the reduction loss is located at the fractured vertebral body without relevant participation of the adjacent vertebral discs.

## Methods

This study used the same cohort of patients that we studied previously [2] with some further inclusion and exclusion criteria. In contrast to the previous study, we only included patients with orthograde beam path of both postoperative and latest lateral radiographs in order to analyze disc and vertebral body heights correctly. Additionally, a posttraumatic MRI was employed to evaluate the potential traumatic intervertebral disc lesions. All inclusion and exclusion criteria are listed in Table 1. This retrospective study was performed at a single level I trauma center between December 2009 and May 2014. The study was approved by the regional ethics committee.

**Table 1:** Inclusion and exclusion criteria

Inclusion Criteria	Exclusion Criteria
Age: > 60 years	Non-orthograde beam path at the fracuted level of the lateral postoperative or final radiographs
Unstable fracture or failed conservative treatment	Inability or unwillingness to join the study
Thoracolumbar junction and mid lumbar spine (Th 11 – L4)	Neurologic impairment
Acute fracture situation	Pathologic vertebral body fractures (tumor/infection)
Posttraumatic total spine MRI	High energy trauma

*Th* Thoracic spine; *L* Lumbar spine; *MRI* Magnetic resonance imaging

A thorough clinical examination, conventional radiographs, and a magnetic resonance imaging (MRI; STIR sequences) of the whole spine were performed on all patients. Patients with MRI contraindications were excluded.

Fracture classification was done in accordance with the new AO spine classification [3], the OF-classification [4], and an OF score of at least 6 [5]. The indication for surgery was in accordance with the spine section of the German society for orthopaedics and trauma (DGOU) [5].

### Surgical techniques

Techniques used were minimally invasive hybrid stabilization by posterior cement-augmented bisegmental instrumentation (Matrix, Fa. DepuySynthes; Viper, Fa. DepuySynthes, Longitude, Fa. Medtronic) using polyaxial screws and without posterior fusion and bilateral transpedicular kyphoplasty (Vertecem, Fa. DepuySynthes) of the fractured vertebral body. All pedicle screws were cement augmented.

### Postoperative management

Postoperatively, all patients received conventional radiographs while standing. No brace or corset was used. Physiotherapy was initiated on the day after surgery to improve mobility and muscle strength. Clinical and conventional radiological assessment was performed regularly.

### Outcome parameters

All patients were evaluated after a minimum of 2 years following initial surgery. Initially the patients were contacted by phone and asked if they were willing to participate. Those patients who participated underwent a clinical examination at the study center including an anterior-posterior x-ray centered on the injured vertebral body and lateral 36 in. views while standing. The vertebral body and the intervertebral discs heights adjacent to the fractured vertebral body between the lateral postoperative radiographs and the lateral radiographs of the final follow-up were compared. Additionally, the height of the disc superior to the instrumentation was used as a reference disc. This was included to analyze the average loss of disc height based on the physiological process of aging. Patients with insufficient postoperative and/or final lateral radiographs, such as non-orthograde beam path, were excluded.

**Fig. 1:**
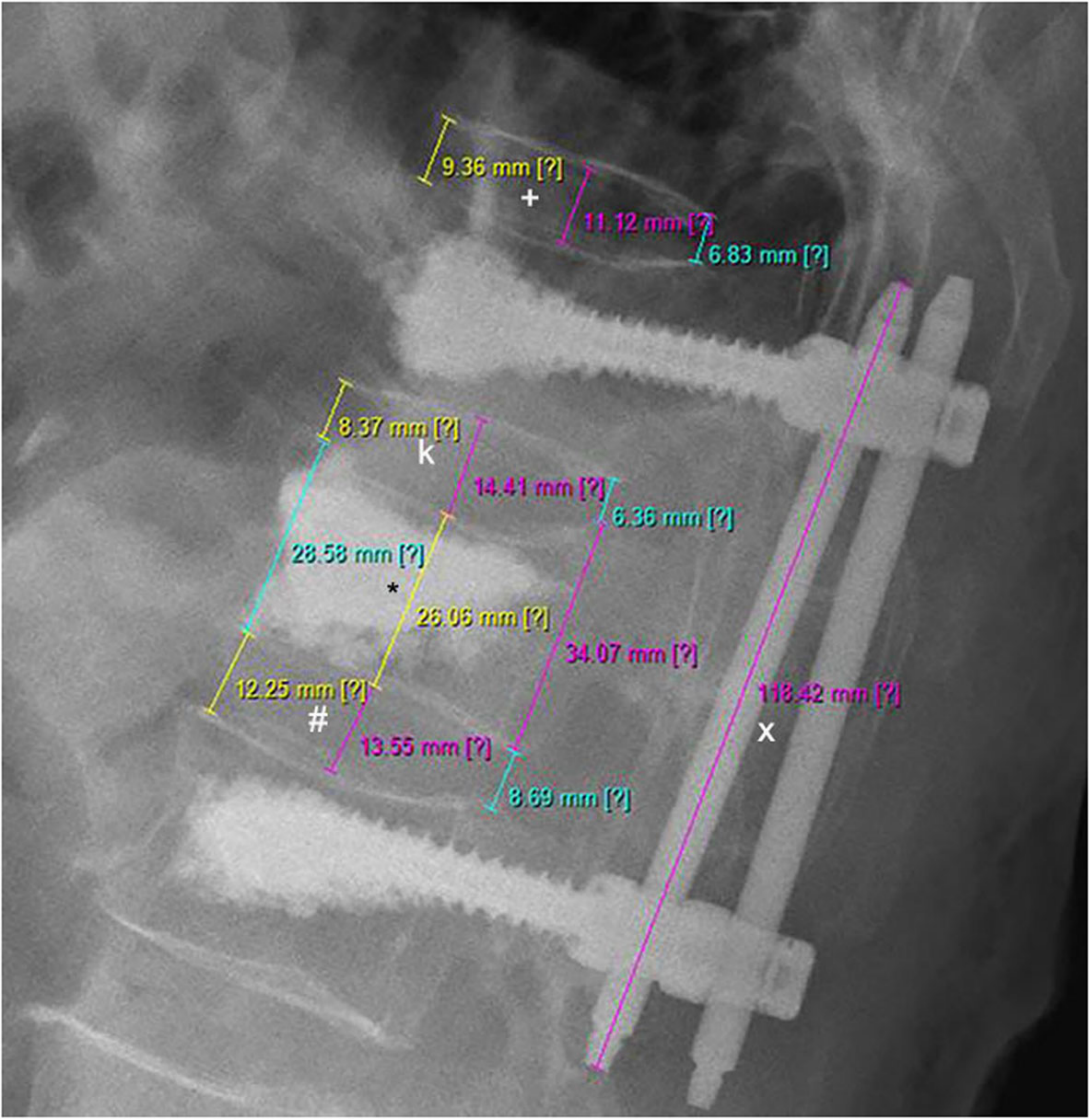
All measurements are depicted. Structures of interests are the fractured vertebral body (*), the superior adjacent intervertebral disc (k), the inferior interverterbral disc (#), the reference disc (+) and the length of the posterior rod (x) in order to minimize magnification errors. The heights of the discs and the vertebral body were measured at the anterior cortex, the posterior cortex and in the center (mid size) between the anterior and posterior cortex. The height of the intervertebral discs were calculated in accordance to Spiegl et al 60] as one third of the sum of the anterior, central and posterior height

**Fig. 2:**
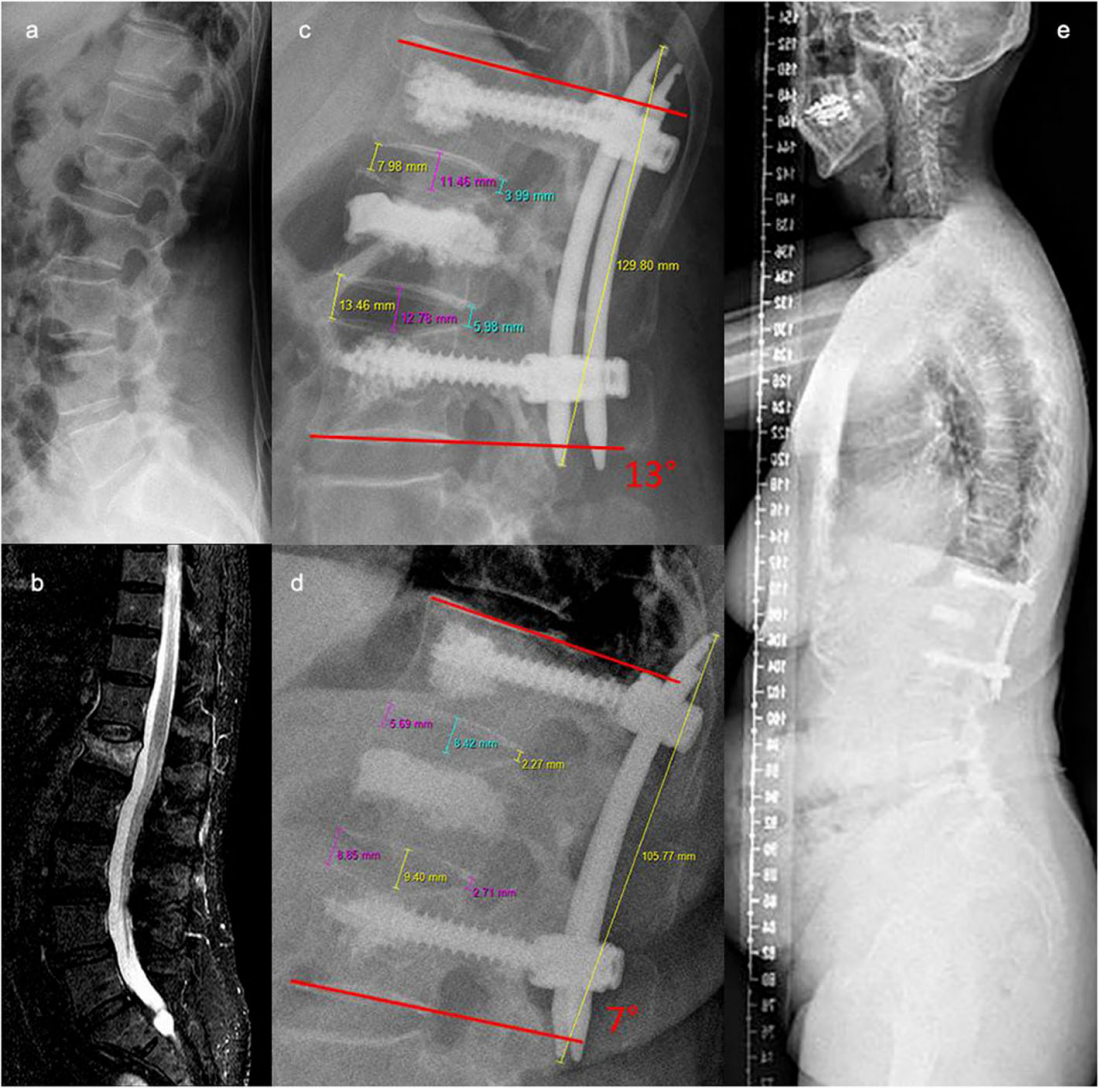
72- year old patient who fell in her apartment while strumbling. An acute fracture of the 2nd lumbar vertebral body with posterior cortex affection of about 25% type OF 3 was diagnosed. Hybrid stabilization was performed after failed non-operative treatment for 7 days with anatomic fracture reduction and a bisegmental lordotic Kobb angle of 13° (c). The latest follow-up were perfomred after 41 months. The patient was very satisfied, suffered from mild pain (VAS: 2) and no limitations (ODI: 0%). A reduction loss of 6 ° is visible (d) with a relative loss of superior disc height of 14% and a compensated sagittal balance (e)

Disc height was defined in accordance with Spiegl et al [6]. The disc height was the average value of three measurements, which were evaluated at three positions; the anterior cortex, the posterior cortex, and at the center between both cortices (Fig. 1). Based on the asymmetric deformity progression of the vertebral body, the vertebral body height was evaluated at the anterior and posterior cortex as well as at the center (Fig. 1). To avoid any magnification errors by the radiographic technique, the implanted rods were used as a reference tool between the postoperative and latest lateral radiographs (Fig. 2).

### Outcome measures

The primary parameters of interests were the relative change of vertebral fracture height (fractured vertebral body) and the disc heights of the intervertebral discs adjacent to the fractured vertebral body as well as the disc superior to the instrumentation, which was used as a reference disc. Implant positioning was also analyzed. Correlations between the relative change in vertebral body and disc heights, the bisegmental reduction loss, as well as all patient parameters were evaluated.

### Statistics

Statistical analysis was performed using standardized SPSS software 17.0 (SPSS®, Inc. Chicago, USA). Statistical analysis was made using descriptive statistics. Fisher’s exact test and paired t-test were used to evaluate any associations between the relative changes in vertebral body heights as well as intervertebral disc heights and the bisegmental reduction loss. Pearson correlation coefficient (coef) was used to calculate correlations between parameters. A significance level of 0.05 was used.

## Results

A total of 29 patients met all inclusion criteria. The average age was 73.3 years (range 61 to 98 years) and the majority of patients were females (72%). Most fractures were located at L 1 (*n* = 11) and Th 12 (*n* = 8), less commonly at L 2 (*n* = 5), L3 (*n* = 3), Th 11 (n = 1), and L 4 (n = 1). Most fractures were incomplete burst fractures, 20 * OF 3 with relevant posterior cortex affection and 6 * OF 2 fractures with minor posterior cortex affection and an OF score of at least 6. Three patients suffered from complete burst fractures (A 4; OF 4). All patients with OF 2 fracture had persistent pain and aquired relevant loss of reduction after mobilization. The average follow-up was 36 months (range: 24–58 months). No obvious trauma-associated intervertebral disc lesions were seen in the posttraumatic MRIs. A total of three patients suffered from adjacent fractures during the follow-up period (10.3%).

### Primary outcome

**Fig. 3:**
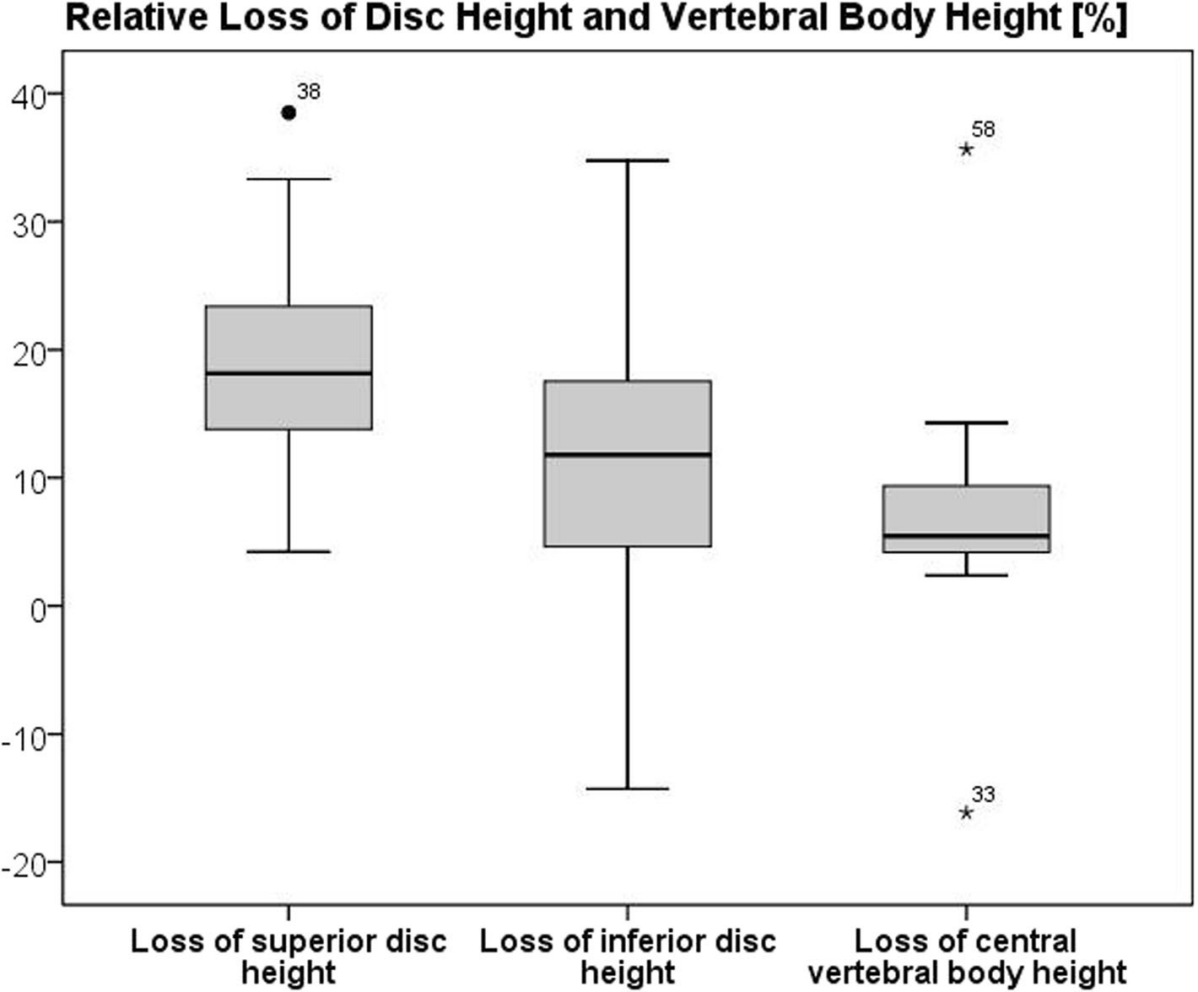
Box plot showing the relative loss of disc height (superior (left) and inferior (middle) ) and the relative loss of height of the central part of the fractured vertebral body during a three year period. Thereby, patient numbers 33, 38, and 58 were negative outlier

The average loss of reduction was 7.7° (range: 1° – 25°). The relative loss of vertebral body and intervertebral disc heights are presented in Table 2 and Fig. 3. The heights of both adjacent intervertebral discs (superior disc: *p* < 0.001; inferior disc: *p* = 0.001), the reference disc (*p* = 0.003) and the central part of the fractured vertebral body (*p* = 0.006) were significantly reduced at the latest follow-up compared to the postoperative radiographs. Thereby, the relative loss of height of the superior adjacent disc was significantly higher compared to the reference disc (p = 0.001). However, only the relative loss of central vertebral body height and loss of reduction had a significant correlation (coef = − 0.471; *p* = 0.048). The changes of the anterior and posterior vertebral body heights were not significant. Generally, all parameters varied widely.

**Table 2:** Changes between the lateral postoperative and latest radiographs

Parameter	Mean	Std	Range
Reduction loss [°]	7.7	5.8	1–25
Rel. loss of disc height: superior [%]	19.0	9.0	4–39
Rel. loss of disc height: inferior [%]	11.9	11.2	-14 – 35
Rel. loss of vertebral body height: central [%]	7,2	9.6	−16 – 36
Rel. loss of vertebral body height: anterior [%]	2.9	8.3	−17 – 21
Rel. loss of vertebral body height: posterior [%]	3.4	10.0	−18 – 36
Rel. loss of disc height: reference disc [%]	6.6	10.9	−22 - 19

*Std* Standard deviation; *Rel* Relative

### Implant position and correlations between the evaluated parameters

No implant failure was visible. No signs of screw cut-out or screw-loosing were recognized. There were no significant correlations between the loss of disc height and the reduction loss, nor between those parameters and the fracture location, fracture classification, age of the patients, gender, and time of follow-up.

## Discussion

The most important finding of this study was that particularly the superior disc adjacent to the fractured vertebral body and the central part of the fractured vertebral body were responsible for the main portion of reduction loss after hybrid stabilization of unstable thoracolumbar fractures. There was a significant correlation between the loss of reduction and the loss of height at the central part of the fractured vertebral body, without any significant correlations between the loss of reduction and the other parameters. Additionally, the highest relative loss of height was seen in the superior disc adjacent to the fracture and it was significantly higher compared to the reference disc. In contrast, the relative loss of the anterior and posterior cortex of the vertebral bodies was low and not significant between the postoperative and the latest radiographs. Furthermore, no implant associated causes for reduction losses could be observed.

Interestingly, the superior adjacent intervertebral disc seems to be responsible for the main portion of reduction loss even though no obvious severe trauma-associated disc lesion was observed on the posttraumatic MRI. However, the posttraumatic MRI consisted of short tau inversion recovery (STIR) sequences only and was not repeated during the further course. Thus, a sufficient analysis of intervertebral disc lesions was not possible [7, 8]. Generally, our patients had a history of only low- to moderate-energy traumata. Posttraumatic disc lesions are rather unlikely in such patients. More than 80% of the fractures were incomplete burst fractures of the superior vertebral body with potential fracture-associated disc involvement. This would explain the significantly higher loss of disc height compared to the reference disc, despite the continual high biomechanical stress of the reference disc superior of the instrumentation [9]. Additionally, the loss of height of both intervertebral discs might be caused by obstructed motion, due to posterior stabilization or cement-associated reduced blood flow to the superior or inferior endplate, leading to a lack of sufficient nutritional supply and resulting in an acceleration of the degenerative cascade [10, 11]. Similarly, Pachowsky et al [12] found increased disc degeneration adjacent to fractured vertebral bodies treated by kyphoplasty. Another hypothesis that would explain the reduction loss is a partial shift of intervertebral disc material into the depression of the centrally fractured vertebral body. This would explain the significant correlation between loss of central vertebral body height and loss of reduction despite comparable anterior and posterior vertebral cortex heights. This might be reduced by optimal cement technique and cement positioning between the upper and middle third of center and the anterior third of the fractured vertebral body. The authors attempted to position the cement at this location of the vertebral body. However, no postoperative computertomography was performed to evaluate the exact cement positioning.

Generally, hybrid stabilization for the treatment of unstable thoracolumbal verterbral body fractures have been reported in several studies [13–16]. The loss of reduction in geriatric patients is reported to be quite high ranging between 4.6 to 23° [17–19]. However, some of the studies did not use cement-augmentation to reinforce pedicle screw stability. We could not detect any form of implant loosening or screw cut-out in our patient population. However, no computer tomography (CT) was done during the final follow-up to rule out screw loosening that might have been missed in conventional radiographs under consideration of the reduced local visualization capacity based on the bone cement. In contrast, the reduction loss in a normal post traumatic osteoporotic scenario can be assumed to be higher. Minamide et al. [20] evaluated 51 patients with comparable osteoporotic vertebral body fractures and comparable age. One group of patients was initially treated non-operatively with kyphoplasty in the further course, whereas kyphoplasty was intially performed in the other group. At the latest follow-up after 1.2 years the local kyhphosis was 28.4° in the delayed kyphoplasty group and 9.5° after initial kyphoplasty.

Arabmotlagh et al [21] just recently reported a complete loss of reduction one year after isolated kyphoplasty with an intervertebral expender. However, the loss of correction over the follow-up period did not correlate with the clinical outcome in their patient population. In contrast, Spiegl et al [2] reported a significant correlation between the patients’ limitations defined by the ODI scores and the amount of reduction loss. In this context, Li et al [22] reported a positive correlation between the amount of lumbar lordosis and fatty infiltration of the low back muscles. This might be a reason leading to a worse clinical outcome. Thus, a persistent re-creation of the anatomic alignment might have positive impact on the clinical outcome, particularly in the long-term. Possible solutions to reduce the amount of reduction loss might be the additional implementation of fracture screws that increase stability or additional posterior fusion [23]. However, an open or a more extended minimally invasive approach would be necessary to perform posterior fusion, leading to longer surgeries and higher blood loss.

The indication for surgery has to be discussed critically in all patients [24]. Some of the patients might have comparable clinical outcomes without surgery or with augmentation procedures of the fractured vertebral body alone. Generally, we have seen the indication for an operative stabilization very strictly and in accordance with the recommendation of the spine section of the German Society of Orthopaedics and Trauma (DGOU) [5]. Surgery was indicated in patients with unstable vertebral fracture and relevant destruction of the anterior column defined by OF scores of three and higher or failed conservative therapy [25]. Additionally, all patients were informed about a non-operative therapy summing up all advantages and disadvantages.

This study offers several limitations. First of all, the retrospective study design has to be discussed critically. Additionally, the patient sample size is rather small. An MRI at the latest follow-up would be desirable in order to get more information on the intervertebral discs adjacent to the fractured vertebral body and the impact of posttraumatic disc lesions on the reduction loss. A posttraumatic MRI including T1 and T2 sequences would improve the quality of disc analysis [7]. Minor screw loosening cannot be completely ruled out, particularly after a cement-augmented pedicle screw implantation technique. A CT examination would be very helpful to analyze the osseous consolidation of the fracture and detect screw loosening that might cause reduction loss. However, the radiation exposure is not justifiable in patients with good clinical outcome. No reference sphere was used. Instead, the posterior stabilization rods were measured in order to ensure identical size relationship. However, coronal malalignment and variation of the beam path could lead to diminished comparability, and a non-orthograde beam path can impede measurement accuracy. Additionally, the time of day at which the examinations took place was not identical. Therefore, smaller disc height could be explained by examinations later in the day that might influence the results. Moreover, no DEXA-scans were routinely done during the admission. This was recommended in the ambulatory setting. Unfortunately, in 41% of the patients (*n* = 11) no osteoporotic screening was performed and only 11 patients received a specific antiosteoporotic therapy at the time of the latest follow-up. This may have influenced the regional alignment. Finally, additional regular radiographic follow-ups (e.g. yearly follow-ups) would be beneficial in order to get information about the time course of reduction loss and loss of disc height.

## Conclusion

The mean reduction loss was moderate 3 years after hybrid stabilization of unstable osteoporotic vertebral fractures of the thoracolumbar spine. A significant loss of both disc heights adjacent to the fractured vertebral body and the central vertebral body height was seen, with the highest loss in the superior disc significantly outranging the reference disc. Thus, the superior disc adjacent to the fractured vertebral body and the central part of the fractured vertebral body seem to be responsible for the majority of reduction loss.

## Data Availability

The datasets during and/or analysed during the current study available from the corresponding author on reasonable request.

## Abbrevations

AO: Arbeitsgemeinschaft Osteosynthese
Coef: Coefficient
CT: Computer tomography
DGOU: German society for orthopaedics and trauma
L: Lumbar vertebral body
MRI: Magnet resonance imaging
ODI: Oswestry disability index
OF: Osteoporotic fracture
STIR: Short tau inversion recovery
Th: Thoracic vertebral body
